# Indoleamine 2,3-Dioxygenase 1 Inhibitor-Loaded Nanosheets Enhance CAR-T Cell Function in Esophageal Squamous Cell Carcinoma

**DOI:** 10.3389/fimmu.2021.661357

**Published:** 2021-03-22

**Authors:** Jingwen Shao, Lin Hou, Jinyan Liu, Yulin Liu, Jie Ning, Qitai Zhao, Yi Zhang

**Affiliations:** ^1^ Biotherapy Center, The First Affiliated Hospital of Zhengzhou University, Zhengzhou, China; ^2^ School of Life Sciences, Zhengzhou University, Zhengzhou, China; ^3^ School of Pharmacy, Zhengzhou University, Zhengzhou, China; ^4^ Henan Key Laboratory for Tumor Immunology and Biotherapy, Zhengzhou, China; ^5^ State Key Laboratory of Esophageal Cancer Prevention & Treatment, Zhengzhou University, Zhengzhou, China

**Keywords:** ESCC, combinatorial immunotherapy, IDO1, CAR-T, epacadostat, hyaluronic acid-modified nanomaterial graphene oxide

## Abstract

In chimeric antigen receptor (CAR)-T cell therapy, the role and mechanism of indoleamine 2, 3 dioxygenase 1 (IDO1) in enhancing antitumor immunity require further study. IDO1 is one of the most important immunosuppressive proteins in esophageal squamous cell carcinoma (ESCC). However, the IDO1 inhibitor, epacadostat, has failed in phase III clinical trials; its limited capacity to inhibit IDO1 expression at tumor sites was regarded as a key reason for clinical failure. In this study, we innovatively loaded the IDO1 inhibitor into hyaluronic acid-modified nanomaterial graphene oxide (HA-GO) and explored its potential efficacy in combination with CAR-T cell therapy. We found that inhibition of the antitumor effect of CAR-T cells in ESCC was dependent on the IDO1 metabolite kynurenine. Kynurenine could suppress CAR-T cell cytokine secretion and cytotoxic activity. Inhibiting IDO1 activity significantly enhanced the antitumor effect of CAR-T cells *in vitro* and *in vivo*. Our findings suggested that IDO1 inhibitor-loaded nanosheets could enhance the antitumor effect of CAR-T cells compared with free IDO1 inhibitor. Nanosheet-loading therefore provides a promising approach for improving CAR-T cell therapeutic efficacy in solid tumors.

## Introduction

The application of chimeric antigen receptor (CAR)-T cell therapy in cancer, following the successful application of CD19 CAR-T cells in eradicating hematologic malignancies, has re-energized cancer immunotherapy research (1, 2). Nonetheless, the solid tumor microenvironment poses many challenges for the application of CAR-T cell therapy (3). To kill solid tumor cells, CAR-T cells must traffic from the blood to solid tumor sites, and then infiltrate the stromal elements to elicit specific cytotoxicity. Their efficacy in solid tumors is constrained by the limited infiltration of immune-cell to the tumor site, heterogeneity of tumor antigen expression, and the immunosuppressive environment (4, 5). Recent efforts have focused on combination therapy to improve CAR-T cell efficacy in solid tumors.

IDO1 mediates the metabolism of tryptophan into kynurenine (KYN), a immunosuppressive metabolite (6). This metabolic pathway creates an immunosuppressive environment in tumors and in tumor-draining lymph nodes (7). The depletion of tryptophan and accumulation of immunosuppressive tryptophan catabolites can induce T cell anergy and apoptosis (8, 9). Importantly, the IDO1 pathway serves as a negative feedback mechanism that is followed by CD8^+^ T cell infiltration (10). Although IDO1 is induced mainly by T cell-mediated IFN-γ, IDO1 overexpression disables T cells (11, 12). IDO1 is produced principally by tumor cells, and is overexpressed in many types of human cancers. Both IDO1 expression by tumor cells and high levels of serum KYN are associated with poor prognosis in patients (13–15). Therefore, tryptophan catabolism has become an attractive target for reducing tumor progression and improving antitumor immunity in cancer therapy (16).

The lymphocyte-dependent IDO1 inhibitor (17), epacadostat (INCB024360), have entered human clinical trials over the years, but all have failed. A recent phase III ECHO-301 trial, testing the combination of epacadostat and pembrolizumab in melanoma, did not show better outcomes than pembrolizumab alone. This led to the halting of other phase III trials of IDO1 inhibitors (18). Since the phase I and phase II trials of epacadostat have shown a good clinical effect (19–21), further study of this drug should not be abandoned. Analysis of the clinical trial results indicates that the dose of epacadostat might be one of the reasons for its failure. Using higher epacadostat doses, to improve target coverage, is an option worth investigating (18, 19, 22, 23). Considering the complex tumor microenvironment in the solid tumors, the ability of the drug to reach the tumor site may be a major reason for IDO1 inhibitor failure in solid tumors; improving this may enhance clinical outcomes.

Graphene oxide (GO) has attracted attention as a promising multi-functional tool with applications in diverse fields, including biomedical engineering (24). Nano-graphene oxide (NGO), which has a large surface area for loading of aromatic drugs, has shown great potential in drug delivery (25). Hence, epacadostat can be easily loaded onto GO nanosheets. Further, many tumor cells show upregulated expression of hyaluronic acid (HA) receptors, making HA a utilized as a capping agent for tumor-specific targeted and controlled drug release (26, 27). Hyaluronic acid-modified graphene oxide (HA-GO) nanosheets have been used in other fields of cancer therapy (27), their ability to enhance CAR-T-induced cell death has not previously been studied.

Therefore, we present the first study evaluating the effects of combining CAR-T cells and IDO1 inhibitors. We combined an IDO1 inhibitor with mesothelin CAR-T cells, using epacadostat-loaded HA-GO nanosheets. This work provides a novel strategy to enhance the efficacy of CAR-T cells in solid tumors.

## Materials and Methods

### Bioinformatics Analysis

Level 3 mRNA sequencing data and clinical information about esophageal cancer were downloaded from UCSC Xena (http://xena.ucsc.edu/). The Spearman correlation between IDO1 and immune-related gene expression in a wide range of solid tumors was downloaded from the cBioPortal for Cancer Genomics (https://www.cbioportal.org) and visualized using a heatmap in R, using the package “pheatmap.” The correlation between IDO1 expression and overall survival in ESCC was calculated using the R packages “survival” and “survminer.” The “high” and “low” IDO1-expression groups were allocated based on the IDO1 expression threshold. Immune-cell abundance was estimated using the R packages “ssGSEA” and “CIBERSORT,” using the supplied cell markers. Samples with *P* < 0.05 were selected for further analysis. Spearman correlation analysis of IDO1 expression and immune-cell abundance was performed using the R package “corrplot.”

### Cell Lines

The human ESCC cell lines (EC109, EC1, TE1, KYSE70, KYSE150, and KYSE450), normal human esophageal epithelial cell line (HET-1A), and human embryonic kidney cell line 293T were purchased from the Chinese Academy of Sciences Cell Repertoire in Shanghai, China,. All cell lines were confirmed free of mycoplasma contamination, and were cultured in DMEM or RPMI-1640 (HyClone, Logan, UT) containing 10% FBS (Sigma-Aldrich), 100 U/mL penicillin, and 100 mg/mL streptomycin, at 37°C with 5% CO_2_. EC1 cells were transduced with a retroviral vector encoding human IDO1 shRNA (EC1-shIDO1) or an empty vector (EC1-control), and with a puromycin-resistance gene. Transduced cells were single-cell cloned by limiting dilution.

### T Cell Isolation and CAR-T Cell Preparation

CD3^+^ T cells from peripheral blood mononuclear cells (PBMCs) were isolated using an autoMACS cell separation device with human CD3 MicroBeads (Miltenyi Biotec). Cells were suspended at a final concentration of 2 × 10^6^/ml in complete RPMI-1640 medium supplemented with 10% FBS, 100 U/mL penicillin, and 100 g/mL streptomycin (28). CD3^+^ T cells were activated using anti-CD3/CD28 conjugated magnetic beads (Invitrogen) at a bead/T cell ratio of 1:1, and then cultured with 100 IU/mL IL-2 (Beijing SL Pharmaceutical, Beijing, China). To generate mesothelin (MSLN)-specific CAR-T cells, we engineered a fusion protein encoding a fully human scFv m912 specific for MSLN (provided by D. Dimitrov), linked to the CD28/CD3ζ domain, as previously described (29).

### Western Blot

Complete cell lysates were clarified by centrifugation and subjected to SDS-PAGE (using 10% polyacrylamide gels). Polyvinylidene fluoride (PVDF) membranes (Bio-Rad, Hercules, CA) were incubated after protein transfer with anti-IDO1 antibody (Adipogen, San Diego, CA), or with anti-β-actin (Cell Signaling Technology) as a loading control.

### Quantitative Real-Time PCR (qPCR)

Total cellular RNA was extracted using TRIzol (Invitrogen, Carlsbad, CA). RNA quality and concentration were detected using a NanoDrop 2000 spectrophotometer (Thermo Fisher Scientific). RNA was reverse transcribed to cDNA using a PrimeScript RT reagent Kit (TaKaRa, Dalian, China). qRT-PCR was performed on a Real-Time PCR System (Agilent Stratagene, Santa Clara, CA), and the data were analyzed by comparative Ct quantification.

### Flow Cytometry and Intracellular Cytokine Staining

Antibodies were purchased from BioLegend. In total, 5 × 10^5^ cells were collected by centrifugation and were washed twice with PBS. The cells were then stained with fluorescence-conjugated antibodies for 20 min in the dark. For analysis of intracellular cytokines, some PBMCs were stimulated with brefeldin (1 3 brefeldin; BioLegend), PMA (1 mg/mL; Sigma-Aldrich), and ionomycin (1 mg/mL; Sigma-Aldrich) for 5 h. Tumor-infiltrating lymphocytes (TILs) from mouse tumors were directly harvested. TILs and stimulated cells were then stained with antibodies against CD45 and CD3 for 20 min on ice in the dark, followed by the addition of 4% formalin. After washing using permeabilization washing buffer, cells were stained with antibodies against IFN-γ, IL-2, and TNF-α for 20 min. Data were acquired on a FACSCanto II flow cytometer (BD Biosciences, Franklin Lakes, NJ).

### Cytotoxicity Assay

CAR-T cells with or without KYN treatment were then cocultured with transduced cancer cells at different effector-to-target (E:T) ratios for 6 h. The tumor cells were then incubated with Annexin-V (BioLegend) for 15 min at 4°C in the dark, and propidium iodide (Sigma-Aldrich) was added before flow cytometry analysis. For the luciferase assay (30), EC1 cells and EC109 cells expressing luciferase (hereafter “luc-EC1 cells” and “luc-EC109 cells”) were treated with PBS, IDO1 inhibitor, IDO1i-loaded nanosheets, and nanosheets for 2 h. Then, 1 × 10^4^ cells per well were placed in a 96-well round-bottom microtiter plate with CAR-T cells, at E:T ratios ranging from 5:1 to 1:1, or alone. After 24 h of coculturing, the supernatant was discarded for ELISA assay. The cells were then transferred into a 96-well black assay plate (Corning 3603) within 50 μL of Dual-Luciferase Reporter Media, followed by incubation at room temperature for 10 min. Fluorescence was then measured using the Xenogen IVIS-200 Spectrum camera imaging system.

### Degranulation Assay

For the degranulation assay, MSLN-CAR-T cells, or KYM-treated MSLN-CAR-T cells, were cocultured with EC1 or transduced EC1 cells for 6 h in complete medium. After stimulation, cells were washed and labeled with anti-CD8 and anti-CD107a antibodies for 20 min at 4°C.

### Preparation and Characterization of HA-GO-IDO1i Nanosheets

HA-GO was synthesized according to our previous report (27). In brief, HA was first aminated (HA-NH_2_) for the reaction with carboxylic acids of GO. Accordingly, HA-NH_2_ was conjugated with GO in the presence of EDC and NHS at room temperature.

For INCB024360 loading, 5 mg of INCB024360 and HA-GO were dissolved in 500 μL ethanol and 3 mL water, respectively. INCB024360 was then added dropwise to the HA-GO solution, and the resulting mixture was stirred at room temperature for 12 h. Finally, the solution was dialyzed against distilled water for 24 h using a dialysis membrane (MW = 12,000 Da) to remove free drugs, and was lyophilized for further use. INCB024360 drug loading was calculated as follows:

*Drug loading (%) = weight of INCB024360 in nanosheets / weight of the whole formulation × 100 %.*

INCB024360 encapsulation efficiency was calculated as follows:

*Encapsulation efficiency (%) = weight of INCB024360 in nanosheets / weight of INCB024360 initially provided × 100 %.*

The resultant formulation of HA-GO-IDO1i was characterized by UV-Vis spectrophotometry, and observed by atomic force microscopy (AFM).

### Cell Proliferation

To determine the effects of the nanosheets on the proliferation of ESCC cells, EC1 cells were seeded in a 96-well plate at a density of 2 × 10^4^ cells/well. After incubating for 1 d, free IDO1i, HA-GO, and HA-GO-IDO1i (at the same IDO1i concentration, 50 μM) were used for treatment for 24 h, 48 h, or 72 h. Then, 10 μL CCK-8 was added to each well, and the cells were incubated for 2 h in a 37°C incubator, and absorbance (OD) was measured at 450 nm using a spectrophotometer. A blank background group, comprising wells with only DMEM medium. Each group had four replicates.

### ELISA

Concentrations of IFN-γ, IL-2, and KYN (all from CUSABIO) were analyzed by ELISA, according to the manufacturers’ instructions. Absorbance at 450 nm was measured on a Molecular Devices Multifilter F5 plate reader.

### *In Vivo* Assays and Quantitative Biodistribution of Nanosheets in Organs

Female NOD-SCID mice (aged 4–6 weeks) were purchased from Beijing Vital River Laboratory Animal Technology Company. Animal care procedures and experiments were approved by the Institute Animal Care and Use Committee of the First Affiliated Hospital of Zhengzhou University (approval number 2019-41). EC1 cells were inoculated subcutaneously into the right flank of female NOD-SCID mice (4–6 weeks old). Tumor volume was estimated as length (mm) × width (mm)^2^/2. When the tumors reached 200–300 mm^3^, the mice were injected *via* the tail vein with 100 μL of IR783, GO-IR783, or HA-GO-IR783 (at the same IR783 concentration, 0.8 mg/kg). The mice were sacrificed 24 h after injection, and the organs were collected for imaging of IR783 fluorescence (720 nm excitation and 790 nm filter), using a Xenogen IVIS-200 Spectrum camera imaging system. Images were acquired and analyzed using Living Image version 4.4 (Caliper Life Sciences, Waltham, MA).

For further *in vivo* assays, NOD-SCID mice were injected subcutaneously with luc-EC1 cells, and developed tumor nodules ∼7 d later; they were then divided randomly into groups. When the primary tumor reached ~50 mm^3^, the mice were subcutaneously injected at their tail-base with 100 μL of PBS, IDO1i, or HA-GO-IDO1i (at the same IDO1i concentration, 100 μg/100 μL). Twelve hours after injection, the mice were infused with 1 × 10^7^ MSLN-CAR-T cells. After 7 d, the phenotypes and cytokines of the CD3^+^ T cells from the tumor tissues were evaluated using flow cytometry. For histopathological analysis, the major organs (liver, spleen, kidney, heart, and lung) were collected and embedded in paraffin. The sections were stained with hematoxylin and eosin (H&E).

### Statistical Analysis

The results were analyzed *via* ANOVA or a one-tailed Mann–Whitney U test, using GraphPad Prism 5.0 (GraphPad Software, La Jolla, CA). The statistical significance threshold was set at *P* < 0.05.

## Results

### IDO1 Accumulation in ESCC May Restrict the Efficiency of CAR-T Cell Therapy

**Figure 1:**
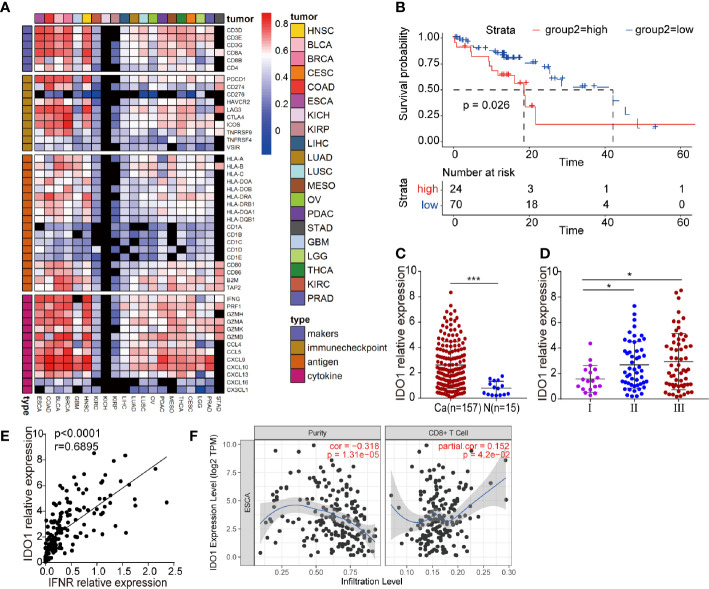
IDO1 expression in ESCC. **(A)** Heatmap showing the correlation between IDO1 and immune-related gene expression in a wide range of solid tumors. Cells present Spearman correlation coefficients (*P* < 0.05); black cells are not significant. **(B)** Correlation between IDO1 expression and overall survival in ESCC. **(C–E)** Correlation between IDO1 expression and clinical prognosis in ESCC. **(E)** Simple linear regression, revealing an inverse relationship between IFNR and IDO1 expression, in ESCC patients from the TCGA database. **(F)** The relationship between IDO1 expression and CD8^+^ T cell infiltration in ESCC, in the TIMER database. *P < 0.05, ***P < 0.001 (repeated-measures one-way ANOVA or Student’s *t-*test).

We studied the correlation between IDO1 expression and the immune signature in multiple solid tumors. IDO1 expression was closely correlated with immune-related gene expression (**Figure 1A**). We therefore focused on examining the function of IDO1 in ESCC. Cancer Genome Atlas (TCGA) sequencing data were used to analyze the correlation of IDO1 expression with the pathological staging and survival of patients with ESCC. Average overall survival was lower in patients with high IDO1 expression than in those with low IDO1 expression (**Figure 1B**). IDO1 expression was significantly higher at tumor sites than in adjacent normal tissues (**Figure 1C**), and IDO1 expression was lower in samples from patients diagnosed at an earlier stage than in those diagnosed at a late stage (**Figure 1D**). This reveals that IDO1 expression was associated with an unfavorable clinical outcome in patients with ESCC, suggesting that the IDO1 pathways might serve as negative feedback mechanisms. As IDO1 is mainly induced by T-cell-mediated IFN-γ, we suggested that expression of IDO1 was significantly positively correlated with the expression of IFNR in ESCC (**Figure 1E**), as well as with the infiltration of CD8^+^ T cell (**Figure 1F**).

**Figure 2:**
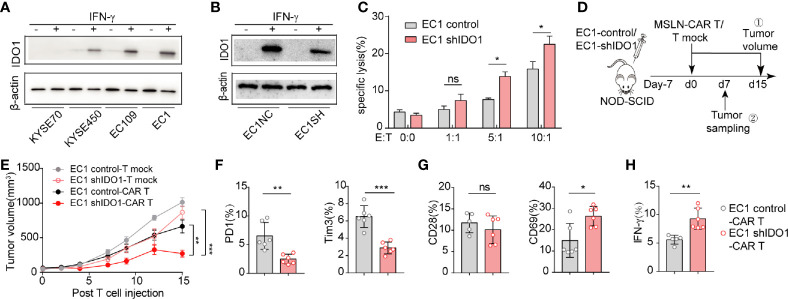
IDO1 accumulation in ESCC may restrict the effectiveness of CAR-T cell therapy. **(A)** Western blot analysis of IDO1 and β-actin levels in ESCC cells treated with or without IFN-γ. **(B)** Western blot analysis of IDO1 and β-actin levels in EC1 cells stably expressing shControl or shIDO1. **(C)** Apoptosis was determined by Annexin-V/PI staining of shControl or shIDO1 cells after 6 h of CAR-T cell culture. **(D)** Schematic of the experiments using SCID/Beige mice, comparing the antitumor effects of MSLN-CAR-T cells in the groups treated with EC1-shControl or EC1-shIDO1 cells. **(E)** Tumor volume was evaluated for 15 d. Expression of **(F)** PD-1,TIM-3, **(G)** CD28, and CD69 in tumor-infiltrated CAR-T cells. **(H)** Proportions of IFN-γ, IL-2, and perforin in tumor-infiltrated CAR-T cells. *P < 0.05, **P < 0.01, ***P < 0.001, ns, Not statistically significant (repeated-measures one-way ANOVA or Student’s *t-*test).

We then assessed whether IDO1 expression affects CAR-T-induced cell death. First, we examined IDO1 expression in the esophageal cancer cell lines: IDO1 expression was highest in EC1, and almost absent from KYSE70 (**Supplementary Figure S1A**). As for IFN-γ induces IDO1 expression in tumor cells, IFN-γ induced IDO1 expression in all lines, except KYSE70 (**Supplementary Figure S1B**, **Figure 2A**). We therefor selected EC1 as the model for further study. The shRNA treatment reduced IDO1 mRNA expression (**Supplementary Figure S1C**, **Figure 2B**). The cytotoxicity of MSLN-CAR-T cells was inhibited in the EC1-control cells, compared with the EC1-shIDO1 cells *in vitro* (**Figure 2C**).

To evaluate the effect of tumor-derived IDO1 on MSLN-CAR-T therapy, we injected EC1-control and EC1-shIDO1 cells into NOD/SCID mice (**Figure 2D**). Seven days later, we injected human non-transduced T cells, or MSLN-CAR-T cells, intravenously. Although both the shIDO1-injected and control tumors grew rapidly after the infusion of human non-transduced T cells, only the shIDO1 tumors were inhibited by MSLN-CAR-T cells. In contrast, the control tumors were resistant to MSLN-CAR-T cell inhibition (**Figure 2E**). The expression of PD-1 and TIM3 was lower in the shIDO1-tumor-infiltrated CAR-T cells than in the control-tumor-infiltrated CAR-T cells (**Figure 2F**). There was no significant difference in CD28 production between the shIDO1 and control tumors (**Figure 2G**). The expression of IFN-g and IL-2 was higher in shIDO1-tumor-infiltrated CAR-T cells than in control-tumor-infiltrated CAR-T cells (**Figure 2H**). These results suggest that IDO1 can inhibit CAR-T cell function in ESCC.

### IDO1 Inhibits Mesothelin CAR-T Cell Function *via* Its Metabolite KYN

**Figure 3:**
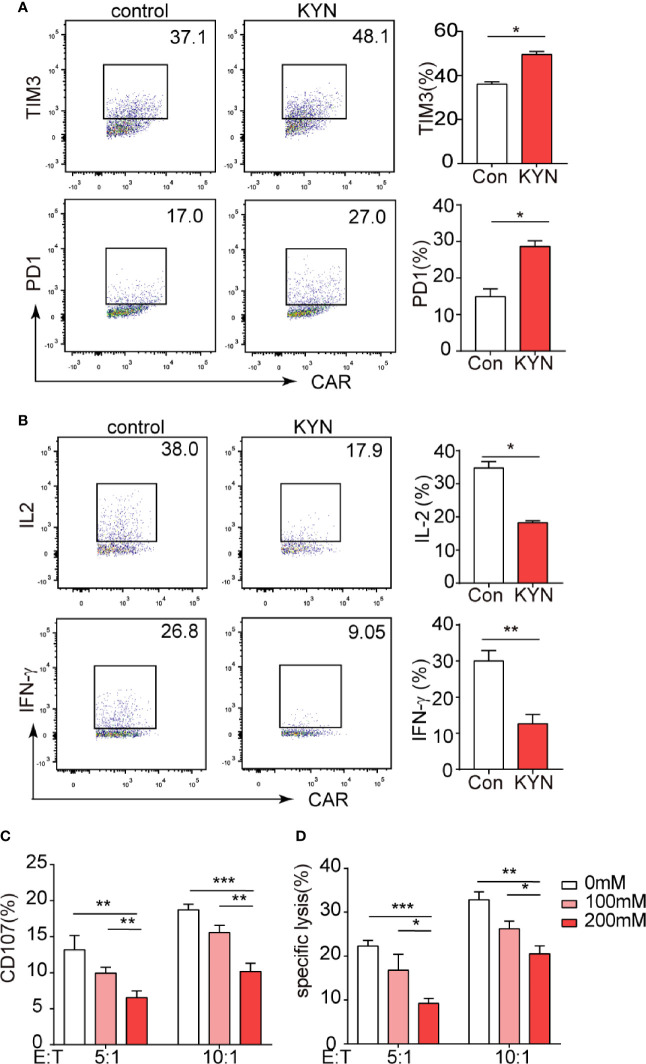
IDO1 can inhibit MSLN-CAR-T cell function *via* its metabolite kynurenine. **(A, B)** MSLN-CAR-T cells were cultured in the absence or presence of KYN (50 μM) with IL-2 (50 U/mL) for 24 h. TIM3, PD-1, IFN-γ, and IL-2 were then detected *via* flow cytometry. **(C)** CAR-T cells treated with different concentrations of KYN were cocultured with tumor cells, to test CAR-T-mediated killing of tumor cells. Tumor cell apoptosis was determined *via* annexin V/PI staining. **(D)** CAR-T cell CD107 expression was detected *via* staining with anti‐CD107a antibody or isotype control antibody, and was analyzed using flow cytometry. *P < 0.05, **P < 0.01, ***P < 0.001 (repeated-measures one-way ANOVA or Student’s t-test).

IDO1 is most widely studied for its role in mediating the metabolism of tryptophan into KYN (31), which was documented inhibits T cell function (32, 33). But the effects of the KYN on CAR-T cells is unknown. We assessed that even at low concentrations (50 µM), KYN significantly promoted PD-1 and Tim-3 expression in CAR-T cells *in vitro* (**Figure 3A**). In contrast, KYN significantly inhibited the expression of the CAR-T cell functional cytokines, IFN-γ and IL-2 (**Figure 3B**). After treatment with KYN, the CAR-T cells were cocultured with IDO1 tumor cells for 4h. Flow cytometric analysis revealed that KYN inhibited MSLN-CAR-T cell cytotoxicity in a dose-dependent manner (**Figures 3C, D**).

### Nanosheet-Based Delivery of IDO1 Inhibitor

**Figure 4:**
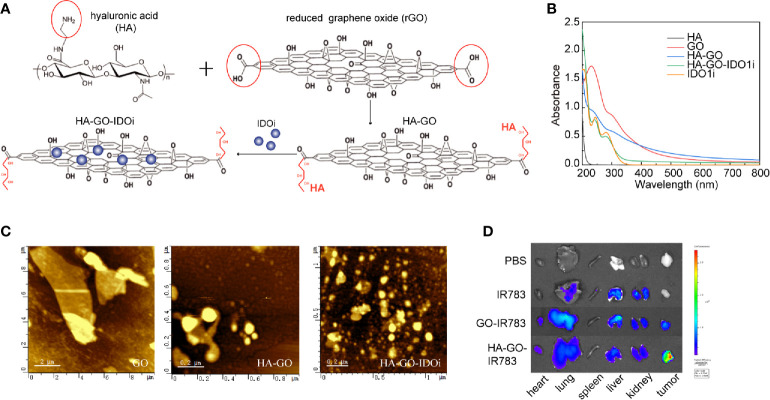
Nanosheet-based delivery system carrying the IDO1 inhibitor. **(A)** Schematic of GO-HA-IDO1i nanosheet synthesis. **(B)** UV-Vis-NIR absorption spectra of the various GO nanosheets. **(C)** Atomic force microscopy images of the various GO nanosheets. **(D)** *Ex vivo* imaging of IR783-labeled HA-GO nanosheets in various organs at 24 h post-injection.

HA-GO-IDO1i nanosheets were synthesized as described (**Figure 4A**). GO showed a broad spectral peak, at 231 nm, and HA was strongly absorbed at 200 nm. After the formation of HA-GO, the absorption red-shifted from 231 to 260 nm, whereas the characteristic peak of HA was maintained, suggesting that the reaction occurred between HA and GO. INCB024360 showed a broad peak at 290 nm. The HA-GO-IDO1i nanosheets also showed a broad peak at 290 nm, suggesting that IDO1i was loaded onto HA-GO (**Figure 4B**). It is possible for IDO1i to be encapsulated into these nanosheets with drug loading as high as 40.0%, mainly *via* π-π stacking, with encapsulation efficiency of 63.2%. The morphologies of the GO, HA-GO, and HA-GO-IDO1i nanosheets were evaluated using atomic force microscopy. After HA modification, the GO particles were smaller and better dispersed. HA-GO-IDO1i remained as nanometer-sized lamellar structures (**Figure 4C**). The fluorescence images of organs from mice sacrificed 24 h post-injection demonstrated the superior tumor-targeting ability of the HA-GO nanosheets (**Figure 4D**). The nanosheets accumulated mainly in the reticuloendothelial system organs (liver, lung, and kidney), as well as in the tumors; the fluorescence was still detectable after 24 h. The fluorescence signals in the liver, lung, and kidney were lower in the HA-GO-injected mice than in the GO-injected mice. These results indicated that HA-GO nanosheets have greater tumor-targeting potential than GO alone, and can enhance the accumulation of GO nanosheets and IDO1i at the tumor site.

### IDO1 Inhibitor-Loaded Nanosheets Mitigate the Inhibitory Effects of Tryptophan Metabolites

**Figure 5:**
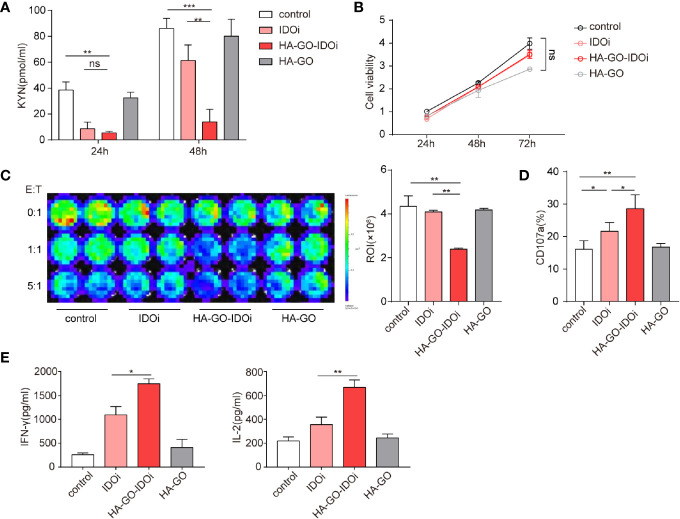
IDO1 inhibitor-loaded nanosheets can mitigate the inhibitory effects of tryptophan metabolites. **(A)** KYN production of EC1 cells cultured with or without IDO1 inhibitor, measured *via* ELISA. **(B)** Effects of nanosheets on ESCC EC1 cell proliferation; **(C)** CAR-T-mediated killing of tumor cells. EC1 cells carrying the firefly luciferase gene were cocultured with CAR-T cells at different ratios, after 24 h of culture with or without IDO1 inhibitor. Luminescence was used to determine percentage tumor cell death. **(D)** CD107a expression of CAR-T cells cocultured with EC1 cells at different E:T ratios for 6 h. The EC1 cells were first cultured with or without IDO1 inhibitor for 24 h. **(E)** Cytokine production of CAR-T cells cocultured with EC1 cells with or without IDO1 inhibitor. IFN-γ and IL-2 secretion of CAR-T cells, detected *via* ELISA. *P < 0.05, **P < 0.01, ***P < 0.001, ns, Not statistically significant (repeated-measures one-way ANOVA or Student’s *t-*test).

Cellular uptake of HA-GO nanosheets was evaluated *in vitro*. Epacadostat is a selective inhibitor which created to bond with the active site of IDO1 enzyme by forming a coordinate covalent bond with ferrous iron of heme (34). KYN production in cells cultured with or without the IDO1 inhibitor was measured *via* ELISA, suppression of KYN production was greatest in the HA-GO-IDO1i group (**Figure 5A**; **Supplementary Figure S2A**). ESCC cell proliferation did not differ significantly between the control group and the groups treated with non-loaded IDO1i or non-loaded nanosheets (**Figure 5B**). We next assessed the effect of the IDO1 inhibitor-loaded nanosheets on CAR-T cell cytotoxicity. After cocultured with luc-EC1 tumor cells that were firstly treated with IDO1i, CAR-T cells showed robust cytotoxicity in HA-GO-IDO1i group (**Figure 5C**, **Supplementary Figure S2B**). CD107 expression was used as another indicator of CAR-T cell cytotoxicity(**Figure 5D**, **Supplementary Figure S3C**). Further, HA-GO-IDO1i treatment significantly inhibited the expression of the CAR-T cell functional cytokines, IFN-γ and IL-2 (**Figure 5E**, **Supplementary Figure S3D**)

### IDO1 Inhibitor-Loaded Nanosheets Protect CAR-T Cells Against Damage by IDO1-Positive Tumors *In Vivo*

**Figure 6:**
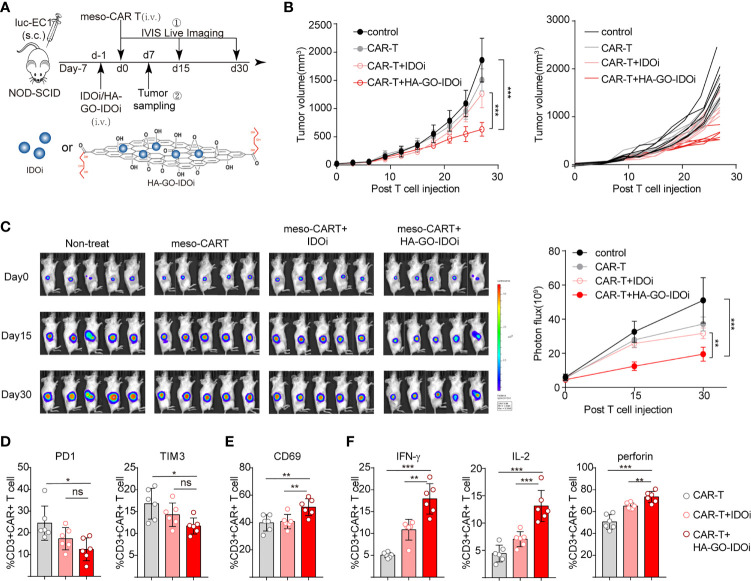
IDO1 inhibitor-loaded nanosheets protect CAR-T cells against the deleterious effects of IDO1-positive tumors *in vivo*. **(A)** Schematic of the experiments using NOD-SCID mice, comparing the antitumor effects of IDO1 inhibitor (IDO1i or HA-GO-IDO1i), MSLN-CAR-T cells, or the combination of both, on IDO1-positive tumors (originating from luc-EC1 cells). **(B)** Tumor volume was evaluated for 30 d. **(C)** Longitudinal measurements of luc-EC1 cell bioluminescence in mice. Expression of **(D)** PD-1, TIM-3, and **(E)** CD69 in tumor-infiltrated CAR-T cells. **(F)** Expression of IFN-γ, IL-2, and perforin in tumor-infiltrated CAR-T cells. *P < 0.05, **P < 0.01, ***P < 0.001, ns, Not statistically significant (repeated-measures one-way ANOVA or Student’s *t-*test).

To evaluate the effect of HA-GO-IDO1i in tumors treated with MSLN-CAR-T cells, we injected luc-EC1 cells subcutaneously, and treated mice with different forms of IDO1i (IDO1i or HA-GO-IDO1i), CAR-T cells, or with both agents. The mice were intravenously injected with the IDO1 inhibitor 24 h before the CAR-T cells were injected (**Figure 6A**). In the MSLN-CAR-T cell group, HA-GO-IDO1i treatment strongly suppressed tumor cell growth (**Figures 6B, C**); importantly, changes in tumor volume revealed that HA-GO-IDO1i was more effective than IDO1i. Further, in the MSLN-CAR-T group, tumor-infiltrated-CAR-T cell expression of PD-1 and TIM3 was lower, whereas that of IFN-γ and IL-2 was higher, in the HA-GO-IDO1i-treated group than in the IDO1i-treated group (**Figures 6D–F**). To assess whether the nanosheets were biocompatible, the major organs (heart, liver, spleen, lung, and kidney) from the four treatment groups were collected 7 d post-injection (**Supplementary Figure S3**). We did not detect any toxicity of the HA-GO-IDO1i nanosheets to mice. Therefore, using nanosheet-loaded IDO1 inhibitors is more effective than direct application of IDO1 inhibitors for promoting CAR-T cell function in treating esophageal cancer. The improvement in CAR-T cell function is mainly manifested by the increased expression of functional molecules and reduced expression of inhibitory molecules.

## Discussion

The importance of IDO1 in limiting the effectiveness of immunotherapy has recently been demonstrated. Instead of directly influencing cancer cells, IDO1 achieves this by powerfully inhibiting the immune system; in particular, it inhibits T cell activity (10, 35). In this study, high IDO1 expression was associated with immune infiltration and poor survival in patients with ESCC. This suggests that the IDO1 pathway might serve as an important negative feedback mechanism. Considering its immunosuppressive activity, targeting IDO1 might benefit patients with a preexisting T cell-inflamed tumor microenvironment. CAR-T cell treatment of hematologic tumors has recently been shown to be highly effective. This has inspired efforts to apply it to solid tumors in the clinic. However, CAR-T cells have shown limited clinical efficacy for treating solid tumors. The immunosuppressive molecules in the microenvironment, including IDO1, present major hurdles for CAR-T therapy in solid tumors. Thus, blocking IDO1 may enhance CAR-T cell cytotoxicity toward solid tumors.

IDO1 has been found to inhibit CAR-T therapy through the action of tryptophan metabolites in hematologic (36) and solid tumors (37). However, the effect of IDO1 on CAR-T therapy in ESCC patients has not previously been studied. In this study, we investigated whether IDO1 expression by ESCC can inhibit the antitumor effect of CAR-T cells *via* its metabolite KYN. Consistent with a previous report (38), KYN inhibited the cytokine secretion and cytotoxic activity of CAR-T cells, suggesting that production of tryptophan metabolites by IDO1 may at least partially underlie resistance to CAR-T therapy in IDO1-positive tumor cells. Since the immunosuppressive function of IDO1 in tumors is well established; research has therefore focused on inhibitors to block its immunoregulatory function (39). Epacadostat, a hydroxyamidine small-molecule inhibitor, potently suppresses tryptophan metabolism, and has been regarded as a potent adjuvant. However, the first phase 3 trial to evaluate its efficacy in combination with pembrolizumab in advanced melanoma showed no evidence that epacadostat improved outcomes (40).

Multiple hypotheses have been improved to explain the discrepancy between the early phase clinical trial success of epacadostat and the failure of the phase 3 (ECHO-301) trial; these include differences between the treatment populations, inappropriately low epacadostat dosing, and incomplete suppression of intratumoral KYN. The phase 3 trial used 100 mg epacadostat, based on the phase I trial, despite higher doses being tolerated and no maximum-tolerated dose being established (41). This dose was chosen as it was deemed that maximal inhibition of IDO1 activity occurred at doses ≥ 100 mg, with no further reduction in peripheral blood KYN at higher doses. Notably, a simultaneous early phase study used 300 mg of epacadostat; the reported models imply a higher likelihood of IDO1 inhibition at this dose (19, 22). These reports suggest that the current dose of epacadostat was not sufficient to reverse the immunosuppressive environment by blocking IDO1. The optimal dose of epacadostat is being studied in ongoing clinical trials.

In order to deliver IDO1 inhibitors to tumors more efficiently, a novel strategy to enhance the upload of epacadostat into tumor sites is required. Given their capacity for controlled and targeted drug release to specific cells, nanomaterials are critical in tumor therapy (42–45). Lu et al. (46)have revealed that INCB24360 delivery *via* a chlorin-based metal-organic framework achieved positive outcomes using combination photodynamic therapy and IDO1 inhibition. In this study, we loaded an IDO1 inhibitor onto an HA-GO nanomaterial; this material delivers the IDO1 inhibitor into cancer cells *via* receptor-mediated endocytosis pathways (27). Our findings demonstrate that a single treatment with HA-GO-IDO1i downregulates KYN production in esophageal cancer cells—the nanomaterial-based delivery system was able to alter the concentration of KYN and tryptophan in the tumor microenvironment before CAR-T infusion. The HA-GO-loaded IDO1 inhibitor reversed IDO1-induced inhibition of CAR-T therapy more effectively than direct application of free IDO1, both *in vitro* and *in vivo*.

In summary, we have demonstrated that IDO1 expression by esophageal tumors inhibits MSLN-CAR-T activity; blocking IDO1 activity reverses the inhibition of CAR-T-induced cell death. Mechanistically, we investigated whether the immunosuppressive function of IDO1 occurs *via* suppression of CAR-T cell cytokine secretion and cytotoxic activity by the IDO1 metabolite KYN. Importantly, the IDO1 inhibitor-loaded nanosheets exhibited greater ability to restore the function of mesothelin CAR-T cells than direct application of free IDO1 inhibitor, both *in vitro* and *in vivo*. These findings demonstrate the importance of blocking IDO1 in restoring the ability of CAR-T cell to kill cancer cells. Further, this study reveals that applying IDO1 inhibitor-loaded nanosheets to treat solid tumors enhances drug influx at the tumor sites. These findings provide a new strategy to enhance the antitumor effect of CAR-T cell therapy, and a promising approach for the treatment of solid tumors.

## Data Availability Statement

Publicly available datasets were analyzed in this study. This data can be found here: http://xena.ucsc.edu/.

## Ethics Statement

The animal study was reviewed and approved by Ethics Committee of the First Affiliated Hospital of Zhengzhou University.

## Author Contributions 

JS, LH, JL, and YZ designed this work and analyzed the data. JS, JL, JN, YL, and QZ helped with, or performed, the experiments and analyses. LH and JN helped to provide the nanosheets. JL, QZ, and LH helped in revising the manuscript. JS and YZ wrote the manuscript. All authors contributed to the article and approved the submitted version.

## Funding

This study was supported by the National Natural Science Foundation of China [Grant No. 82001659], the Program of the Major Research Plan of the National Natural Science Foundation of China [Grant No. 91942314], and the National Science and Technology Major Project of China [Grant No. 2020ZX09201-009].

## Conflict of Interest

The authors declare that the research was conducted in the absence of any commercial or financial relationships that could be construed as a potential conflict of interest.
