# Supplementary material for: Indoleamine 2,3-Dioxygenase 1 Inhibitor-Loaded Nanosheets Enhance CAR-T Cell Function in Esophageal Squamous Cell Carcinoma
Source: Front Immunol. 2021 Mar 22;12:661357. doi: 10.3389/fimmu.2021.661357 (PMC8019778; doi:10.3389/fimmu.2021.661357)
Supplement: Supplementary file 1 [file DataSheet_1.pdf]

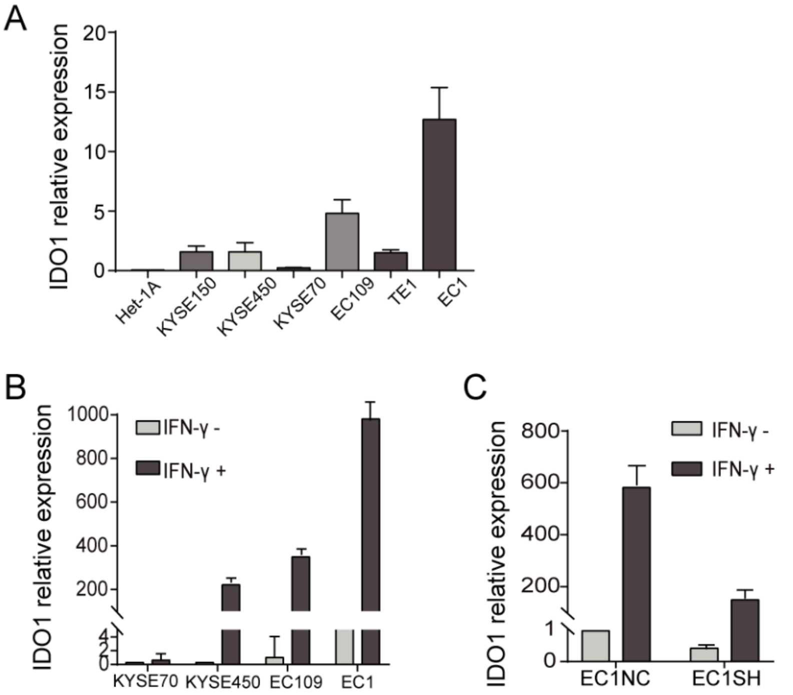


**Supplementary Figure 1** IDO1 mRNA levels, measured using qPCR. ESCC cell lines were cultured in the absence or presence of 50 U/mL exogenous IFN-γ for 24 h.

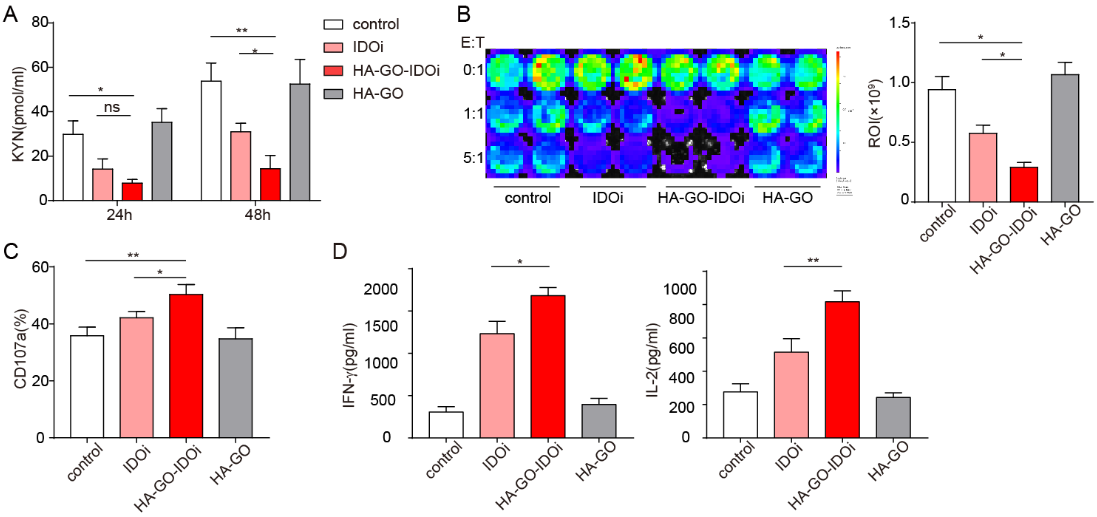


**Supplementary Figure 2.** (A) KYN production of EC109 cells cultured with or without IDO1 inhibitor. (B) After 24h treaded with or without IDO1 inhibitor, Luc-EC109 cells were cocultured with CAR-T cells at different ratios, Luminescence was used to determine percentage tumor cell death. (C) CD107a expression by CAR-T cells cocultured with IDO1 inhibitor-treated EC109 cells. (D) IFN-γ and IL-2 secretion of CAR-T cells cocultured with IDO1 inhibitor-treated EC109 cells., detected via ELISA. *$P < 0.05$, **$P < 0.01$, ***$P < 0.001$ (repeated-measures one-way ANOVA or Student's *t*-test)

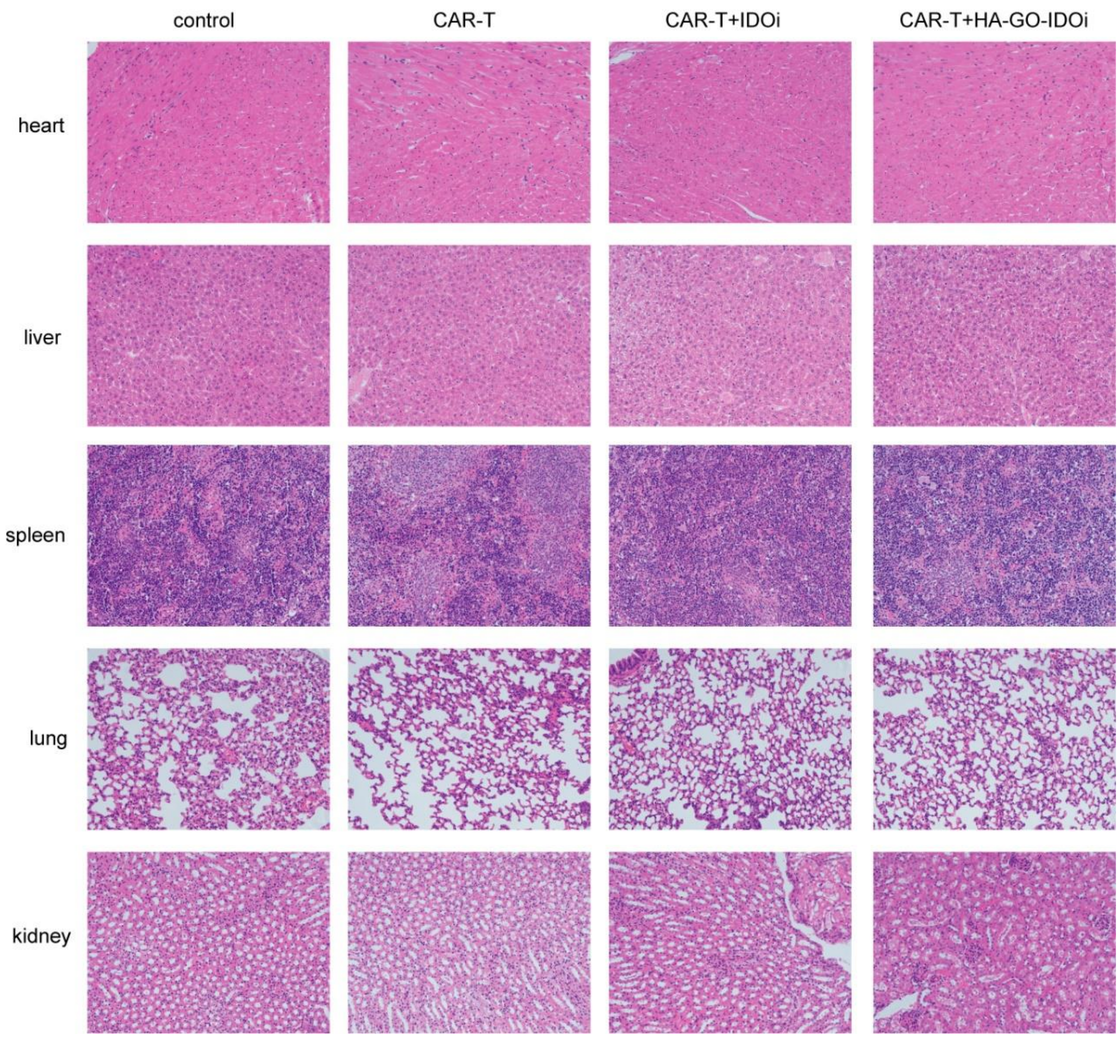


**Supplementary Figure 3.** H&E-stained images of slices of the major organs, collected from treated mice 7 d post-injection.
